# Improving the Postmarket Surveillance of Total Joint Arthroplasty Devices

**DOI:** 10.2174/1874312900802010007

**Published:** 2008-02-25

**Authors:** Nizar N Mahomed, Khalid Syed, Clement B. Sledge, Troyen A Brennan, Matthew H Liang

**Affiliations:** 1 Division of Orthopaedic Surgery, Toronto Western Hospital, University Health Network, University of Toronto, 399 Bathurst St., ECW 1-002, Toronto, Ontario, Canada M5T 2S8; Clinical Epidemiology and Health Care Research Program, Department of Health Administration, University of Toronto, Canada; 2 Department of Orthopedic Surgery, Brigham and Women’s Hospital, Harvard Medical School, Boston, MA, USA; 3 Division of Internal Medicine, Brigham and Women’s Hospital, Harvard Medical School, Boston, MA, USA; 4 Division of Rheumatology, Immunology and Allergy, Brigham and Women’s Hospital, Harvard Medical School, Boston, MA, USA; Section of Rheumatology, VA Boston Health Care System, USA

## Abstract

**Objective:**

To evaluate the FDA’s approval process and postmarket surveillance strategies for THR devices.

**Design:**

The FDA Center for Devices and Radiological Health (CDRH) 510k releasable database was used to document approved THR devices. The CDRH Medical Device Reporting data files were used to study the efficiency of the FDA’s post-market surveillance system. Manufacturers were contacted to supply information regarding their implants. Medline was searched between 1966-1996 to determine the percentage of THR devices with published data on clinical outcomes.

**Results:**

Between 1976 and 1996, 701 new THR devices were approved by the Substantial Equivalent (SE) route and 34 were approved on the basis of Premarket Approval PMA. The number of approvals doubled between 1991-1995 compared to 1976-1990. Seventy-four different manufacturers obtained approval to market THR devices. Only four manufacturers obtained approval *via *the PMA application. Under Mandatory Device Reporting all revision arthroplasties should be reported. Using data from 2 independent services for which we had US hospital discharge data in 1993 we estimate that only 3% of all revision THR were reported to the FDA. Manufacturers of hip implants failed to provide useful information. Medline search revealed only 15% of the approved THR devices had published data on outcomes.

**Conclusion:**

Current FDA premarket approval and postmarket surveillance strategies fail to provide information for evidence-based selection of THR devices. Recommendations are made to avert problems with device failures.

## INTRODUCTION

Orthopedic surgery plays a critical role in the management of many forms of arthritis. Total joint arthroplasty, in particular, dramatically improves function and quality of life in persons with end-stage hip and knee disease and its outcomes are generally durable[1]Nevertheless, acute and chronic implant failures occur resulting in accelerated revision surgery. Examples of these include the rapid wear of previous new polyethylenes, such as carbon impregnated polyethylene, heat pressed polyethylene, and hylamer which did not improve outcomes and caused many accelerated revisions [2]. Attempts at improved designs such as the ACS cup which was associated with excessive wear and ultimately led to recall and replacement [3]. A recent and widely publicized example was the Sulzer acetabular cup, Interop, which was recalled in 2000 after 17,000 implants because of acute failed arthroplasties from residue on the cup from the final cleaning process of the porous coating, a change in the manufacturing process from a previously successful APR cup [4].

The withdrawal of rofecoxib (Vioxx^®^) in September, 2004 and the ensuing discussion over the effectiveness and capacity of the Food Drug Administration (FDA) to protect the public safety [5-7] highlights again the challenges of post-marketing surveillance. Despite these mishaps post marketing surveillance of devices has had little or no study.

There are an estimated 200,000 primary hip replacements performed annually in the US[8] with a direct medical cost of total hip arthroplasty estimated at 2 billion dollars annually. All arthroplasties have a finite life and may need revision [9]. The majority of revisions are due to loosening of THR devices and most of these can be directly attributed to their design characteristics [9]. In 1993 there were over 26,000 revisions performed in the US[10]. A tremendous research effort developing devices with improved survival and a competitive industry has resulted in an exponential increase in the number of THR devices being brought to market. Given the rapid expansion of this technology, we examined the FDA medical device approval process and postmarketing surveillance during a period (1976-1995) where there was available information on approved devices and sought the corresponding data on the clinical effectiveness and patient-oriented outcomes of specific devices.

## MEDICAL DEVICE REGULATORY PROCESS

### Pre-Market Approval

In the United States the Food and Drug Agency (FDA) regulates medical devices. Currently there are more than 1700 different types of medical devices, 70,000 different products for specific applications, and 7,000 firms who have FDA approval to market these products[11]. The FDA has a sophisticated and stringent review process for pharmaceutical agents and its policies for medical devices have attempted to replicate these standards to ensure their safety and effectiveness.

In 1976 FDA was given the authority to regulate medical devices under the Medical Device Amendment Act (MDA). This Act focused on the premarket review process and contained four key elements including classification, three-tier controls, premarket notification system, and comparable regulation of old and new devices. Under classification the FDA categorized all existing and newly approved devices into seven (non mutually exclusive) categories including pre-amendment device (devices that were on the market prior to 1976), post-amendment device (devices brought to market after 1976), substantially equivalent device (post-amendment device that is equivalent to a pre-amendment or approved post-amendment device)[11] (Table **1**). Prior to 1976 there were a handful of total hip implants available. These were classified as pre-amendment devices all pre-amendment hip devices were grandfathered under the MDA and allowed to remain on the market.

The three-tier control system grouped devices into three categories depending upon the least amount of regulation necessary to provide reasonable assurance of safety and effectiveness (Table **2**). Class I devices are required only to be registered with the FDA. Class II devices require manufacturers to provide data on their design, material properties, and mechanical characteristics. Class III devices require manufacturers to file a Premarket Approval application (PMA) which includes data from a controlled clinical trial. The distribution of pre-amendment devices into the three-tier system included 30% as Class I, 60% as Class II, and 10% as Class III [11].

The third element of the MDA was a premarket notification system, which provided a mechanism for evaluating post-amendment devices. This requires manufacturers to notify the FDA prior to marketing any device not previously marketed. Notification consists of a 510(k) application, which describes the new device; it’s material and design characteristics, and intended use. The FDA reviews the 510k to determine if the device is “substantially equivalent” to an existing approved device and thereby giving it clearance for marketing. If the device is judged not substantially equivalent, the manufacturer is required to submit a PMA application prior to marketing. The PMA requires the manufacturer to submit data addressing questions of material biocompatibility, mechanical performance, biological function, and a controlled clinical trial comparing the performance of the proposed device with that of a control device[11, 12].

The fourth element of the MDA was the principle that new devices should not be disadvantaged by the gradual implementation of the MDA requirements. This allowed any post-amendment device that was substantially equivalent to either a pre-amendment or approved post-amendment device to be marketed until the FDA either established standards or demanded a formal PMA application for Class III devices. It was not until the late 1980’s that the FDA started to address the backlog of Class III pre-amendment devices and their substantially equivalent post-amendment counterparts for which PMA submissions were required to prove clinical safety and effectiveness[11]. Effectively this allowed pre-amendment and post-amendment substantially equivalent total hip devices to be marketed without providing any clinical performance data until the FDA issued a call for PMA submissions. In the case of total hip devices the FDA issued this call in September 1996.

In 1992 the FDA Commissioner at that time, Dr. David Kessler, formed the Committee for Clinical Review (CCR) to review the medical device approvals process of the agency[11]. The CCR reviewed 24 PMA and 510(k) applications that were pending review or had been approved. They concluded that the clinical data provided for approval (1) failed to utilize appropriate controls (2), used poorly defined historical controls (3), used sample sizes inadequate to answer questions (4), poorly characterized study subjects (5), poorly assessed the comparability of patients in treatment and control groups (6), failed to clearly and consistently define study endpoints, and (7) failed to have blind evaluation of subjective endpoints.

## Post-Market Surveillance

In 1993 Commissioner David Kessler summarized his agency’s position on the need for postmarket surveillance and that the safety and effectiveness of medical devices over a long period of time, in day-to-day use in unselected patients with comorbid conditions, without a protocol follow-up of a formal clinical study could not be guaranteed with even the most stringent premarket approval process[13]. Initially the FDA’s primary strategy for post market surveillance was the Problem Reporting Program (PRP), a completely voluntary system by which health professionals and consumers could notify the FDA of device related problems. Under-reporting of adverse events plagued the system and to improve postmarket surveillance Congress enacted the Safe Medical Devices Act (SMDA) in 1990. The SMDA established the Mandatory Device Reporting program (MDR), which mandated reporting of death and serious injuries to the FDA by a nationwide sample of facilities specially trained to collect complete and accurate data on device-related products to the Medical Product Surveillance Network (MEDSUN) [14]. “Serious injury” defined under MDR includes all events necessitating a return to the operating room related to a medical device failure. Under this definition nearly all revision THR should be reported to the FDA although most would judge late revisions (1020 years out from the initial surgery) very differently than early revisions which are sentinels of problems with the device. With the current reporting system, the surgeon doing the revision may not know the surgeon, the hospital, or the implant associated with the original surgery.

## METHODS

### Evaluation of Premarket Approval Process

To evaluate the pre-market approval process, a Freedom of Information request was submitted to the FDA to identify and describe the total number of hip replacement devices that had been approved from 1976 to 1995. It asked for all hip prosthetic devices listed under CFR21 subchapter H medical devices Part 888 orthopedic devices subpart D prosthetic devices number 888.3300 – 888.3410. For each device we requested the name, its manufacturer, the FDA classification (Class I, II or III), method of FDA pre-market approval (510k or PMA), and date of approval. Second, we inspected the FDA Internet web site at http:\\www.fda.gov for all medical devices approved by the FDA between 1976 to 1995 by 510k or PMA applications. This database lists all devices by date of approval, device category, device name, its manufacturer, and method of approval (510k *vs* PMA). The data from these two sources were combined for this analysis.

### Evaluation of Postmarket Surveillance Process

To evaluate the effectiveness of the MDR postmarket surveillance system we obtained data from two independent sources from 1993 for which we had US hospital discharge data. First, we obtained from the FDA, the number of adverse events reported to the MDR program for 1993. Second, we used the National Hospital Discharge Survey for 1993 to obtain the number of revision total hip replacements performed in 1993[10]. The number of MDR reports were compared to the number of revision surgeries in 1993 to define the adverse event capture rate of the MDR system.

### Evaluation of Available Clinical Data

To assess the availability of data on the clinical outcomes of hip replacement devices we contacted the manufacturers identified in the pre-market approval evaluation exercise that had received approval between 1976 to 1995 for marketing a total hip device. Each manufacturer was asked to provide the names of all total hip devices they had marketed, a description of the device, details of the surgical technique, FDA classification, date of marketing, and any published data on the clinical outcomes of their device.

Second, to identify published information on the outcomes of total hip devices we conducted a Medline search of publications from 1966 to 1996 using the key word “hip prosthesis” and text words for all possible combinations of total hip device trade names and/or names of manufacturers. Reports of in-vitro biomechanical testing of total hip devices were excluded. We were unable to search for devices approved by the PMA route, as the description of these implants by the FDA did not include their trade names. For devices approved by the 510k processes we were able to systematically search for outcomes information by manufacturer and number of approved devices.

## RESULTS

### Pre-Market Notification And Approval Process

**Fig. (1):**
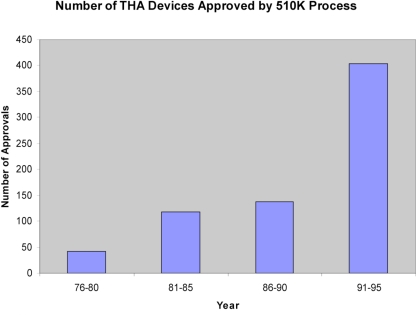
THR devices approved by year, 1976-1995.

Between 1976-1995, 71,239 new medical devices were approved *via *the 510k notification process for devices that were developed post-MDA. Of these, 701 were prosthetic hip devices. Between 1976-1980 42 THR devices were approved, reflecting the relative infancy of the technology at that time (Fig. **1**). During the 80’s there was a steady increase in the number of approvals. Between the years 1981-1985, 118 devices were approved compared to 137 between 1986-1990. By far the largest number of approvals occurred between 1991-1995 when 404 devices were approved. This represents nearly 60% of all approvals in the last 20 years. All of these approvals were on the basis of a new device being substantially equivalent to an existing approved device. By satisfying this requirement, manufacturers were not required to provide clinical data on the safety and effectiveness of their device.

Seventy-four firms developed the 701 devices approved by the 510k process (Table **3**). Of these 52 firms had fewer than 10 approvals each, representing 25% of the total number of approved devices. Thirteen firms held between 10-19 approvals each, representing 24% of all approved devices. Nine firms held 20 or more approvals each representing over 50% of all approved devices.

Between 1976-1995, only 34 hip devices were approved *via *a PMA application that requires submission of clinical data from a controlled clinical trial (Table **4**). Four firms obtained these approvals, in contrast to 74 that held 510k approvals. Each of these 4 firms held more than 20 approvals by the 510k process. One firm had obtained 20 of the 34 PMA approvals. The PMA approval process accounted for less than five percent of all THR device approvals. Clearly the 510k application process is the preferred process by manufacturers as it avoids the need for expensive clinical trials.

## POSTMARKET SURVEILLANCE

The FDA’s MDR system requires that manufacturers, user facilities, physicians, and manufacturers report any device-related deaths, serious injuries, and malfunctions. In the definition of serious injury revision total hip replacements are reportable  events. In  1993  a year in which we had  2 independent sources of data (see methods) the MDR program received 677 reports of prosthetic hip device related problems. The National Hospital Discharge Survey documented over 26,000 cases of revision THR for the same calendar year 1993. Most experts in the field agree that implant failure due to aseptic loosening results from implant related problems and accounts for well over 50% of all revision THR (other indications for revision surgery include infection, technical errors, joint dislocations, etc.). Based on a conservative estimate that 50% of all revision THR are due to implant failure, in 1993 there were over 13,000 reportable cases (due to device related failure) and by our estimates, the MDR picked up only 6% of all reportable cases and underscore the limitations of passive surveillance systems.

## INFORMATION AVAILABLE ON TOTAL HIP DEVICES

To evaluate the information available to physicians and patients regarding the safety of implants from all companies that had obtained FDA approval to market THR devices in the US we sought information regarding their devices, only six firms replied with partial descriptive information about their products. No published reports were provided. Our Medline search of approved THR devices are shown in Table **5**. Overall, only 15% of the 510k approved devices had any published data on clinical outcomes. Twenty-three percent of approved devices from firms with fewer than 10 approvals each had published reports on clinical outcomes. Surprisingly, the proportion of devices with published reports from firms with 10-19 approvals and those with 20 or more approvals each were 17% and 11%, respectively. The vast majority of the published literature were case series from tertiary referral centres where the device was developed. There were no standardized methods of evaluating baseline patient characteristics nor standardized outcomes. In addition results were evaluated unblinded and used various unvalidated physician reported hip scoring systems and not patient-oriented outcomes.

## DISCUSSION

Between 1976-1995 the FDA approved over 95% of all hip replacement devices without requiring any clinical data on the safety or clinical effectiveness of the device. The MDR program, the FDA’s primary postmarket surveillance strategy for medical devices, captures only 6% of the reportable THR device-related problems. This is similar to the report by Castro *et al*. who estimated that less five percent of total joint related complications were reported to the FDA[15]. This low rate of capture highlights the inability of this country’s strategy to ensure safety and effectiveness of these devices. The vast majority of THR devices between 1976-1995 were approved and marketed with little oversight by the FDA. Attempts to obtain clinical data regarding THR devices from manufacturers were unsuccessful. Medline search revealed 15% of 510k approved THR devices had published data on clinical effectiveness. This is consistent with a British study by Murray *et al* that noted fewer than 30% of available THR prostheses in the United Kingdom had any published clinical data[16]. Furthermore, similar to previous reports we found the majority of published data was of poor quality and not useful for critical comparison of individual devices [17, 18].

The results of this study beg the question, is the current system of medical device regulation adequate to ensure the safety and effectiveness of medical devices? In the case of THR devices and we suspect all total joint arthroplasty devices mandating a more stringent premarket approval process by the PMA route would not ensure the long term safety and effectiveness because the required duration of follow-up in FDA clinical trials for is two years. This is inadequate for devices which have service lives extending beyond 10 years. To streamline the premarket approval process, the FDA has reclassified the majority of THR devices from Class III to Class II[19]. As Class II devices, existing and new THR devices do not need to provide clinical data on safety and effectiveness prior to obtaining FDA approval. This policy makes postmarket surveillance even more important to ensure the long term safety and effectiveness of these devices.

The MDR postmarket surveillance system is ineffective, and the 1997 FDA Modernization and Accountability Act (FDAMA) have further restricted the ability of the FDA to conduct postmarket surveillance of medical devices. Under FDAMA the agency is no longer able to mandate postmarket surveillance of implantable medical devices[19]. The FDA must now seek voluntary support from manufacturers in order to have them conduct postmarket surveillance studies, which are restricted to a maximum follow-up of 3.5 years. Consequently, the FDA in 1998 rescinded device tracking requirements for 14 device categories [19]. Overall, the FDA’s postmarket surveillance strategies are more limited now than in the early 1990’s under the SMDA. AdvaMed, the medical device industry lobbying group has opposed reform of the current FDA approval process for devices.

In comparison to the US situation, in Canada, medical device licensing is regulated by Health Canada’s Health Products and Food Branch (HPFB). There are more than 22,000 pharmaceutical products and 40,000 medical devices available in Canada [21]. All medical devices manufactured in or imported into Canada require an application to Health Products and Food Branch. The items are classified based on their risk to the population into one of four classes. Class I devices do not require a license. Classes II to IV are granted licenses based on the application submitted which must include the purpose of the device as well the safety profile and proof of evidence of safety and effectiveness of the device. This is usually in the form of a randomized controlled trial. Health Canada requires medical device manufacturers to use a quality system certificate as evidence of compliance to the appropriate regulatory quality system requirement for manufacturing. Although Health Canada monitors products for failure and adverse effects, this is based on the reporting by manufacturers, health care professionals and consumers of the products and inspections of manufacturing plants and import shipments. In Canada and the United States, there is no specific requirement for the manufacturer to conduct post market surveillence [20].

The increasing restrictions on the FDA premarket approval and postmarket surveillance point to the need for alternative strategies to ensure the safety and clinical effectiveness of these devices. One solution would be the establishment of a national THR registry. Such a registry exists in Sweden, which is 1/20 the size of the US and has a single payor health care system with almost complete electronic linkage of health care data [22]. Although a registry is an appealing option, it would be prohibitively expensive to establish and maintain. It would also require significant alterations in the way medical information is collected and shared between various health care sectors and most importantly it would have to be mandatory not passive. These data would have to be linked to patient identifying information by a unique number, across different states, health care providers, physicians and health care institutions where patients receive care in violation of current HIPPA standards. Finally, it would have to include standardized data and be longitudinal for the life of the patient and/or the device to be useful.

We propose a novel solution which would be mandatory and a condition of approval, where an independent body would plan, oversee, and interpret the results from this system. Key stakeholders would be non-voting members from orthopedic device manufacturers, orthopedic surgeons. Experts in outcomes measurement, statisticians, epidemiologists with no conflict of interest would be paid members.

The system would use standardized criteria for evaluation including minimum number of subjects to insure adequate numbers of subjects after natural attrition and drop outs to provide statistically stable estimates of rare and common adverse events. The system would be financed by all manufacturers in the form of evaluation fees. Devices that meet minimum standards for safety and effectiveness at specified follow-up intervals could be so acknowledged. This could be a powerful incentive for the industry which could use the information in their marketing of a device (for example, as having achieved milestones of longevity and/or outcomes. The body could be responsible for the dissemination of this information to patients, providers and payors (with appropriate protection for manufacturers against product liability actions).

To make tracking specific, reimbursement for both primary and revision arthroplasty should be linked to detailed information about the implant determined à priori. All implant components would be uniquely bar coded or similarly identified and tracked as a condition of payment for primary arthroplasty and when retrieved and/or replaced in revisions. The tracking system would also make surveillance systems using claims data as has been suggested [23] more informative linking outcomes to specific designs.

The success of this system would depend in part on the perceived need by manufacturers to voluntarily report clinical performance data. This goal would be facilitated by support from patients, medical care providers, and payors who would benefit from an evidence-based approach to medical device selection. If large purchasers of health care were to require the use of only accredited implants in their organizations, the manufacturers would be more likely participate in the program. This form of postmarket surveillance could provide an efficient method for evaluating the performance of THR devices without increasing the regulatory requirements of the FDA. Furthermore it would not delay the introduction of new technologies while at the same time improving the quality and level of information available on these devices.

This approach could be applied to any medical device. This scheme might add costs to the FDA but it might not since this system which replaces one that doesn’t work and which has untold costs for patients who suffer ill consequences. In summary, we have studied the premarket notification approval process and postmarket surveillance of the most common surgical device used in the management of patients with arthritis and musculoskeletal diseases and find that it is not working. Data on patient-oriented outcomes or on failed prostheses for specific designs is virtually non-existent. We believe the same is true for other orthopedic implants. We propose a system to correct this. In the mean time, the public should be aware.

## Figures and Tables

**Table 1:** Medical Device Classification System

Categories	Description
Pre-amendment	devices that were marketed prior to May 28, 1976 (the date the MDA was signed into law)
Post-amendment	devices that were approved by the FDA after the MDA was enacted
Substantially Equivalent	devices that are substantially equivalent to pre-amendment devices based on their design, materials, and intended use
Implant	devices that are inserted into a surgically formed or natural body cavity and intended to stay for at least 30 days.
Custom	devices generally not available to other licensed practitioners and not available in finished form
Investigational	devices undergoing clinical investigation under the authority of an Investigational Device Exemption from the FDA
Transitional	devices which were regulated as drugs prior to the Medical Device Amendments but are since regulated as devices

**Table 2:** Three Tier Device Control System

Class	Description
I	devices for which General Controls including manufacturer registration and product listing with the FDA are adequate to ensure safety and effectiveness
II	devices for which General Controls are inadequate and special controls(i.e. standardized testing protocols, bench-testing or clinical data) are required to provide assurance
III	entirely new post-amendment devices, or devices with a new design, or new intended use, or devices found Not Substantially Equivalent (NSE) to pre-amendment devices for which there is insufficient information to assure their safety and effectiveness

**Table 3:** Distribution of THR Devices Approved by 510k Process Between 1976-1995

Number of Approvals Held by Manufacturer	Number of Approved Devices	Number of Manufacturers
0 < 10	174 (25%)	52 (70%)
10 to 19	169 (24%)	13 (18%)
≥ 20	358 (51%)	9 (12%)
Total	701	74

**Table 4:** Distribution of THR Devices Approved by PMA Between 1976-1995

Number of Approvals Held by Manufacturer	Number of Approved Devices	Number of Manufacturers
0 < 10	4 (12%)	2 (50%)
10 to 19	10 (29%)	1 (25%)
≥ 20	20 (59%)	1 (25%)
Total	34	4

**Table 5:** 510k Approved THR Devices with Published Clinical Data

Number of Approvals Held by Manufacturer	Number of Approved Devices	Number of Devices with Published Data	Approved Devices with Published Data (%)
0 < 10	174	40	23%
10 to 19	169	29	17%
≥ 20	358	38	11%
Total	701	107	15%
